# Factors mitigating the harmful effects of intimate partner violence on adolescents' depressive symptoms—A longitudinal birth cohort study

**DOI:** 10.1002/jcv2.12134

**Published:** 2023-01-25

**Authors:** Dawid Gondek, Gene Feder, Laura D. Howe, Ruth Gilbert, Emma Howarth, Jessica Deighton, Rebecca E. Lacey

**Affiliations:** ^1^ UCL Great Ormond Street Institute of Child Health London UK; ^2^ Department of Population Health Sciences University of Bristol Bristol UK; ^3^ Centre for Academic Primary Care University of Bristol Bristol UK; ^4^ MRC Integrative Epidemiology Unit University of Bristol Bristol UK; ^5^ Department of Public Health and Primary Care University of Cambridge Cambridge UK; ^6^ School of Psychology University of East London London UK; ^7^ Evidence Based Practice Unit University College London Anna Freud National Centre for Children and Families Clinical, Educational and Health Psychology London UK; ^8^ Research Department of Epidemiology and Public Health University College London London UK

**Keywords:** adolescence, ALSPAC, birth cohort, domestic abuse, interpersonal violence, protective factors

## Abstract

**Background:**

Preventing parental intimate partner violence (IPV) or mitigating its negative effects early in the lifecourse is likely to improve population mental health. However, prevention of IPV is highly challenging and we know very little about how the mental health of children exposed to IPV can be improved. This study assessed the extent to which positive experiences were associated with depressive symptoms among children with and without experience of IPV.

**Method:**

This study used data from the Avon Longitudinal Study of Parents and Children, a population‐based birth cohort. After excluding those without information on depressive symptoms at age 18, the final sample comprised 4490 participants. Parental intimate partner violence (physical or emotional cruelty reported by mother or partner) when the cohort child was aged 2–9 years. Depressive symptoms were measured with the Short Mood and Feelings Questionnaire (SMFQ) at age 18 years.

**Results:**

Each additional report of parental intimate partner violence (over six reports) was associated with 0.047 (95% CI 0.027–0.066), or 4.7%, higher SMFQ score. Conversely, each additional positive experience (over 11 domains) was linked with −0.042 (95% CI −0.060 to −0.025) or 4.1%, lower SMFQ score. Among those with parental intimate partner violence (19.6% of participants), relationship with peers (effect size = 3.5%), school enjoyment (effect size = 1.2%), neighbourhood safety and cohesion (effect size = 1.8%) were associated with lower levels of depressive symptoms.

**Conclusions:**

Most positive experiences were linked with lower levels of depressive symptoms regardless of parental intimate partner violence exposure. However, among those with parental IPV, this association was found only for relationships with peers, school enjoyment, neighbourhood safety and cohesion on depressive symptoms. If our findings are assumed to be causal, nurturing these factors may mitigate the harmful effects of parental intimate partner violence on depressive symptoms in adolescence.

**Key points**

- We tested to what extent positive experiences were associated with depressive symptoms in adolescence among those who were previously exposed to intimate partner violence.
- Parental intimate partner violence experienced in childhood was associated with more depressive symptoms at age 18. Most positive experiences, such as relationships with parents, teachers, peers, school enjoyment, were linked with fewer depressive symptoms at age 18.
- Among the 19.6% exposed to parental intimate partner violence, this association was found only for relationships with peers, school enjoyment, neighbourhood safety and cohesion on depressive symptoms.
- Interventions aiming to nurture positive relationships with peers, school experiences and neighbourhood safety and cohesion have the potential to improve adolescent depression regardless of exposure to parental intimate partner violence.

## INTRODUCTION

Intimate partner violence (IPV) refers to any behaviour within an intimate relationship that causes physical, psychological or sexual harm (Breiding et al., 2015). IPV is highly prevalent, with between 17% and 24% of women experiencing at least one episode of IPV in their lifetime (Ali et al., 2021). IPV has been consistently found to have a strong link not only with mental health of victims of IPV (Devries et al., 2013), but also their children (Bauer et al., 2013; Bevilacqua et al., 2021). As around half of all common mental disorders, such as depression or anxiety, have their onset in early life (Kessler et al., 2007), preventing IPV or mitigating its negative effects early in the lifecourse is likely to improve population mental health. However, we know very little about the factors that may potentially offset the harmful influences of parental IPV on the mental health of their children.

Although parental IPV is associated with an increased risk of common mental disorders among children, most children exposed to parental IPV do not suffer from mental ill‐health (Gondek et al., 2021). While preventing IPV ought to remain a priority, it is important to understand whether nurturing certain positive experiences, through policies or interventions, can improve the mental health of children already exposed to IPV (Hughes et al., 2018). Emerging evidence on factors alleviating the relationship between IPV, or other childhood adversities, and mental health in children tends to be limited to the use of cross‐sectional design, retrospectively reported information and convenience or unrepresentative samples (Afifi et al., 2016; Afifi & MacMillan, 2011; Bellis et al., 2017; Cheung et al., 2017; Eisenberg et al., 2007; Hu et al., 2015; Hughes et al., 2018; Latham et al., 2021). The most consistent findings emerging from these studies point towards protective effects for mental health of stable and supportive family relationships, positive community and school experiences, particularly for children with severe adverse child experiences (Afifi et al., 2016; Afifi & MacMillan, 2011; Bellis et al., 2017; Cheung et al., 2017; Eisenberg et al., 2007; Hu et al., 2015; Hughes et al., 2018; Latham et al., 2021).

In our study, we used the Health Outcomes from Positive Experiences (HOPE) framework to identify factors that may be linked with lower depressive symptoms in adolescents exposed to early life adversities (Sege & Harper Browne, 2017). HOPE specifies four core domains within which positive experiences are needed to promote health and well‐being. These include nurturing and supportive relationships, safe and protective environments, constructive social engagement and connectedness, and learning social and emotional competencies (Sege & Harper Browne, 2017). We took advantage of the longitudinally and prospectively collected data from birth until 18 years old from the Avon Longitudinal Study of Parents and Children (ALSPAC) study to examine the extent to which positive experiences are associated with symptoms of depressive symptoms among those with or without exposure to IPV.

## METHODS

### Data

This study used data from ALSPAC—a population‐based birth cohort from the Avon region of South–West England. ALSPAC initially recruited 14,541 pregnant mothers with estimated due dates between April 1991 and December 1992 (Boyd et al., 2012; Fraser et al., 2013; Northstone et al., 2019). After bolstering the initial sample with eligible cases that failed to join the study originally, the total sample is 15,454 pregnancies resulting in 15,589 known foetuses. 14,901 were alive at 1 year of age. Further details of the design of ALSPAC can be found elsewhere (Boyd et al., 2012; Fraser et al., 2013). Ethical approval was obtained from the ALSPAC Ethics and Law Committee and the Local Research Ethics Committee. Informed consent was provided from all participants or their carers for each data collection. The study website contains details of all the data that is available through a fully searchable data dictionary and variable search tool: http://www.bristol.ac.uk/alspac/researchers/our‐data/.

## MEASURES

### Parental intimate partner violence

Parental intimate partner violence (or IPV) included instances of being physically or emotionally cruel to the partner (with either mother or her partner being a victim) that were self‐reported via questionnaires by mothers and their partners (see Table S1 for more details). IPV was measured on six occasions when the study children were aged from 2 years 9 months to 9 years, with the recall period at each occasion ranging from 12 to 17 months. To capture the cumulative exposure to IPV, the number of times IPV was reported was summed up, resulting in a count variable ranging from 0 to 6.

### Positive experiences

Using the HOPE framework, we identified 11 domains of positive experiences, each mapping to one of the components of HOPE, comprising (1) nurturing and supportive relationships; (2) safe and protective environments; (3) constructive social engagement and connectedness; and (4) learning social and emotional competencies (Sege & Harper Browne, 2017). The four‐factor structure of the HOPE model was recently validated using a representative sample of Australian children, with the framework having good predictive validity of child health and academic outcomes (Guo et al., 2021).

We were interested in positive experiences that can be targeted by potential policy or interventions, as occurring after parental IPV was identified, to potentially alleviate the association between IPV and adolescents' depressive symptoms. Table S1 show more detailed information about measures of positive experiences used here. Positive experiences were reported by study children, or their parents when study children were between around 9 and 14 years of age and included warm parenting, relationship with parents, co‐parenting alliance, relationships between parents, relationship with teachers, relationship with peers, home education environment, out‐of‐home activities with parents, out‐of‐home activities without parents, school enjoyment, neighbourhood safety and cohesion. Individual research items corresponding to each positive experience were summed up resulting in continuous variables, with a higher score indicating greater exposure to a positive experience. We also derived an overall exposure to positive experiences variable. To do that, we first derived a binary indicator of having a high positive experience, defined as having a score in the top 25th percentile for each domain. Subsequently, we summed up the instances of positive experience across all 11 domains, resulting in the count of total positive experiences with a possible range from 0 to 11.

### Adolescent depressive symptoms

Depressive symptoms were measured with the Short Mood and Feelings Questionnaire (SMFQ; Messer et al., 1995), which was administered to study children at a clinic assessment at around 18 years. The SMFQ includes 13 items that measure the presence of depressive symptoms in the last 2 weeks. The response scale for each question includes “not true” (scored 0), “sometimes” (scored 1) and “true” (scored 2), with the total score of summed items ranging from 0 to 26, where a higher score represents higher levels of symptoms.

### Potential confounding factors

Potential confounding factors were chosen a priori, defined as variables that were likely to be associated with the IPV, positive experiences and depressive symptoms, and were not on the causal pathway between these variables (VanderWeele, 2019). They included—all measured at birth—child's sex, family's ethnicity, maternal partnership status, maternal and paternal education, maternal and paternal social class, financial difficulties, housing tenure, crowding index, mother smoking during pregnancy, mother drinking alcohol during pregnancy, mother's age at birth, parental mental health problems.

## STATISTICAL MODELLING

### Analytic strategy

Firstly, we examined the main effects of IPV and each positive experience (separately), as well as their count (ranging from 0 to 11), on depressive symptoms in adolescents in both crude and confounding‐adjusted models. This analysis helped us to determine whether positive experiences were linked with lower depressive symptoms across the entire sample, regardless of IPV exposure. As recommended (Nicols, 2010), due to positive skewness of depressive symptoms, and inflated proportion of zero scores (*n* = 307, 6.8%), we used generalised linear model (GLM), with a log link function (assuming Poisson distribution) and robust standard errors.

Secondly, we tested the extent to which each positive experience (separately), as well as their count, was associated with depressive symptoms in adolescence among those with and without IPV. We ran separate confounding‐adjusted GLMs for each positive experience, as well as the overall count of positive experiences—separately among those who ever and never experienced IPV. The key estimands of interest for our study aim were the coefficients quantifying the strength of the association between positive experiences and depressive symptoms among those who ever experienced IPV. As a secondary objective, we compared the strength of the association between positive experiences and depressive symptoms between those with and without IPV. This was done by including an interaction term between IPV and each positive experience (e.g., IPV*warm parenting) in adjusted GLMs, with the exposure to IPV serving as a potential effect modifier. This can help us understand whether certain factors may have equal or more beneficial effects among those with IPV than in general population of adolescents.

All analyses used sex‐combined samples to maximise the statistical power necessary to detect interaction effects, and sex differences were beyond the scope of the study. To facilitate interpretability of the key effects, the estimates were also given as a percentage difference after converting beta coefficients using a formula [exp(*b*) − 1] × 100. All analyses were conducted using Stata 17 (StataCorp, 2017). The analysis code is available online at https://osf.io/gesy9/.

### Missing data

Missing data were replaced using multiple imputation by chained equations to minimise the bias due to non‐response, resulting in 50 imputed samples (Sterne et al., 2009; White et al., 2011). In line with recommendations, the imputation model included all the variables from the analyses, as well as interaction terms between IPV and positive experiences (e.g., IPV*warm parenting), ensuring that the relationship between the variables of interest was preserved (Sterne et al., 2009; White et al., 2011). According to the best practice, we deleted imputed outcome (depressive symptoms at age 18) before analysis (von Hippel, 2007). More details about the approach to missing data can be found in Appendix S1 (including Tables S2 and S3).

## RESULTS

### Missing data

The present study includes information from birth until the study children were 18 years old. After excluding those without information on depressive symptoms at age 18, the final sample comprised 4490 participants (see Figure S1), which is 30.1% of the full ALSPAC sample (*n* = 14,901 children alive at 1 year of age).

In the full available ALSPAC sample, the distribution of IPV was highly comparable to the study sample. Likewise, the distribution of positive experiences did not substantially differ in the full available ALSPAC and study samples (see Table S4 for characteristics of the full available ALSPAC sample). Nonetheless, the study sample was socioeconomically more advantaged than the full sample, with, for instance, 19.9% of mothers having a degree in the study sample compared with 12.9% in the full sample. There is also a lower proportion of non‐white individuals in the study sample (4.2% vs. 5.1% in the full sample).

Measures of parental intimate partner violence at individual time points were missing in between 14.9% and 19.3%, and of positive experiences between 16.0% and 27.5%, with neighbourhood safety and cohesion missing in 49.2% (see Table S2). Those with missing data tended to have higher depressive symptoms, have parental IPV, be socioeconomically disadvantaged, be non‐white, have more mental health problems in childhood, have a mother who smoked or drank alcohol during pregnancy and have parents with mental health problems (see Table S3). The association between IPV and greater depressive symptoms, and between positive experiences and lower levels of depressive symptoms were somewhat stronger in the unadjusted models using complete cases (*n* = 1005), compared with the main analysis using imputed data (see Table S5 for estimates based on complete cases).

### Characteristics of the sample

The mean depressive symptoms in adolescence in the study sample, as measured by SMFQ, was 6.59 (standard deviation = 5.25) (see Table 1). 18.4% participants experienced IPV at least once (with 7.6% at least twice). The mean of individual positive experiences ranged from 2.83 to 37.50 on their respective scales, with the average count of total positive experiences (across domains) being equal to 3.23 (SD = 1.94). Most of the participants were white (95.8%), with 4.2% being non‐white. There was a greater proportion of females (57.6%, vs. 42.4 males) in the sample.

**TABLE 1:** Descriptive information about the studied variables—among those with complete measure of depressive symptoms (*n* = 4490)

Variable (age in years when measured)	*N* total	Mean	SD	Min	Max
Outcome					
Depressive symptoms (18)	4490	6.59	5.25	0	26
Exposures					
Parental intimate partner violence (1.5–11)	2917	0.36	0.88	0	6
Total positive experiences (9–5–14)	1195	3.23	1.94	0	9
Warm parenting (12)	3556	13.57	3.27	0	16
Co‐parenting alliance (12)	3588	3.17	1.87	0	6
Relationships between parents (12)	3255	25.76	8.49	0	36
Relationship with parents (9.5)	3513	37.50	3.76	8	40
Relationship with teachers (11)	3761	12.61	3.06	0	25
Relationship with peers (10.5)	3774	7.90	1.32	0	9
Positive home education environment (11.5)	3403	13.02	6.38	0	43
Out‐of‐home activities with parents (11.5)	3426	8.25	3.41	0	25
Out‐of‐home activities without parents (13)	3692	2.83	2.69	0	14
School enjoyment (11)	3428	22.23	5.15	0	30
Neighbourhood safety and cohesion (14)	2282	22.35	3.92	5	30
Confounding factors					
Financial difficulties (0)	3998	2.32	3.18	0	15
Mother's age during birth (0)	3973	29.78	4.53	17	45

	*N* total	*N*	%		
Child's sex	4490				
Female		2587	57.6		
Male		1903	42.4		
Family's ethnicity	4057				
White		3885	95.8		
Non‐white		172	4.2		
Maternal marital status (0)	4163				
Never married		562	13.5		
Widowed/divorced/separated		189	4.5		
1st Marriage		3156	75.8		
2nd/3rd marriage		256	6.1		
Maternal education (0)	4112				
Degree		818	19.9		
A level		1167	28.4		
CSE/Vocational/O level		2127	51.7		
Paternal education (0)	4015				
Degree		1053	26.2		
A level		1146	28.5		
CSE/Vocational/O level		1816	45.2		
Maternal social class (0)	3683				
I/II		1472	40.0		
III Non‐manual/III manual		1624	44.1		
IV/V		587	15.9		
Paternal social class (0)	3335				
I/II		1667	50.0		
III Non‐manual/III manual		1458	43.7		
IV/V		210	6.3		
Housing tenure (0)	4127				
Mortgaged/owned		3509	85.0		
Not owned		618	15.0		
Crowding index (0)	4088				
≤ 0.5		2133	52.2		
>0.5–0.75		1224	29.9		
>0.75–1		584	14.3		
>1		147	3.6		
Mother smoking during pregnancy (0)	4163				
No		3498	84.0		
Yes		665	16.0		
Mother drinking during pregnancy (0)	4082				
No		1185	29.0		
Yes		2897	71.0		
Parental mental health problems (0)	4114				
No		3760	91.4		
Yes		354	8.6		

Abbreviations: A level, advanced level; CSE, certificate of secondary education; O level, ordinary level; SD, standard deviation; 95% CI, 95% confidence interval.

### Main effects of IPV and positive experiences on depressive symptoms

There was strong evidence for the association between parental IPV as well as total positive experiences (i.e., a count of instances when a child scored in the top 25% within domain) and depressive symptoms at age 18, in both confounding unadjusted and adjusted models (see Table 2 for estimates). In adjusted models, each exposure to IPV was associated with 0.047 (95% CI 0.027–0.066), or 4.7%, higher SMFQ score. Conversely, each additional highly positive experience (over 11 domains) was linked with −0.042 (95% CI −0.060 to −0.025) or 4.1%, lower SMFQ score.

**TABLE 2:** Estimates of the association between each exposure and depressive symptoms at age 18

Exposures (range; age in years when measured)	Unadjusted estimates (all participants)		Adjusted estimates^a^ (all participants)		Adjusted estimates^a^ (participants with IPV)		Adjusted estimates^a^ (participants without IPV)	
	*b*	(95% CI)	*b*	(95% CI)	*b*	(95% CI)	*b*	(95% CI)
Parental intimate partner violence (0–6; 1.5–11)	0.060	(0.040, 0.080)	0.047	(0.027, 0.066)	–	–	–	–
Total positive experiences (0–11; 9.5–14)	−0.039	(−0.057, −0.022)	−0.042	(−0.060, −0.025)	−0.039	(−0.071, −0.007)	−0.040	(−0.058, −0.021)
Warm parenting (0–16; 12)	−0.007	(−0.014, 0.001)	−0.007	(−0.014, 0.001)	−0.012	(−0.027, 0.002)	0.000	(−0.009, 0.009)
Co‐parenting alliance (0–6; 12)	−0.027	(−0.041, −0.014)	−0.017	(−0.031, −0.004)	0.001	(−0.028, 0.030)	−0.013	(−0.030, 0.003)
Relationships between parents (0–36; 12)	−0.005	(−0.008, −0.002)	−0.003	(−0.006, 0.000)	0.001	(−0.004, 0.006)	−0.003	(−0.006, 0.001)
Relationship with parents (8–40; 9.5)	−0.010	(−0.017, −0.004)	−0.012	(−0.018, −0.006)	−0.007	(−0.018, 0.004)	−0.013	(−0.020, −0.006)
Relationship with teachers (0–25; 11)	−0.017	(−0.026, −0.009)	−0.021	(−0.029, −0.013)	−0.014	(−0.029, 0.002)	−0.023	(−0.032, −0.014)
Relationship with peers (0–9; 10.5)	−0.019	(−0.038, 0.001)	−0.027	(−0.046, −0.008)	−0.036	(−0.067, −0.005)	−0.020	(−0.043, 0.003)
Positive home education environment (0–48; 11.5)	−0.004	(−0.008, 0.001)	−0.004	(−0.008, 0.000)	−0.006	(−0.014, 0.002)	−0.002	(−0.007, 0.002)
Out‐of‐home activities with parents (0–32; 11.5)	−0.013	(−0.021, −0.005)	−0.010	(−0.018, −0.002)	−0.013	(−0.029, 0.003)	−0.007	(−0.017, 0.002)
Out‐of‐home activities without parents (0–14; 13)	0.001	(−0.009, 0.011)	−0.001	(−0.011, 0.009)	−0.013	(−0.032, 0.007)	0.003	(−0.009, 0.014)
School enjoyment (0–30; 11)	−0.014	(−0.019, −0.010)	−0.017	(−0.021, −0.012)	−0.012	(−0.021, −0.003)	−0.018	(−0.024, −0.013)
Neighbourhood safety and cohesion (3–30; 14)	−0.026	(−0.034, −0.019)	−0.023	(−0.031, −0.016)	−0.018	(−0.032, −0.004)	−0.024	(−0.032, −0.015)

Abbreviations: b, Poisson beta coefficient; IPV, intimate partner violence; 95% CI, 95% confidence interval.

^a^ Adjusted confounding (all measured at birth): child's sex, family's ethnicity, maternal partnership status, maternal and paternal education, maternal and paternal social class, financial difficulties, housing tenure, crowding index, mother smoking during pregnancy, mother drinking during pregnancy, mother's age at birth, parental mental health problems.

Out of all the 12 studied individual positive experiences, 10 were found to be associated with depressive symptoms in confounding‐adjusted models: warm parenting, co‐parenting alliance, relationship between parents, relationship with parents, relationship with teachers, relationship with peers, positive home education environment, out‐of‐home activities with parents, school enjoyment, neighbourhood safety and cohesion. Their effect sizes ranged between 0.1% and 2.7% SMFQ score per one unit on their respective scales (see Table 2 for the exact values of coefficients).

### Effects of positive experiences of depressive symptoms—Stratified by IPV exposure

Among the 19.6% with IPV exposure, there was evidence for an association between total positive experiences and depressive symptoms in confounding‐adjusted models. Each additional positive experience was linked with −0.039 (−0.071, −0.007) or 3.8%, lower SMFQ score. Three of the individual positive experiences were associated with depressive symptoms in those with IPV in confounding‐adjusted models: relationship with peers (effect size = 3.5%), school enjoyment (effect size = 1.2%), neighbourhood safety and cohesion (effect size = 1.8%) (see Table 2 for coefficients).

Among those without IPV, there was an association with depression in confounding‐adjusted models for relationship with peers (effect size = 2.0%), teachers (effect size = 2.3%), parents (effect size = 1.3%), school enjoyment (effect size = 1.8%), neighbourhood safety and cohesion (effect size = 2.3%).

The estimates of interaction effects between parental IPV and any of the studied positive experiences (or their count) were highly unstable, as shown by wide confidence intervals of their interaction terms (see Table 3 for exact values of the coefficients). Hence, we did not find any strong evidence for differences between those with and without IPV in the association of positive experiences (or their count) with depressive symptoms.

**TABLE 3:** Adjusted estimates of an interaction effect between each positive experience and parental intimate partner violence, with depressive symptoms at age 18 as an outcome

	*b*	(95% CI)
Total positive experiences	−0.041	(−0.059, −0.024)
Parental IPV	0.028	(−0.020, 0.076)
IPV*total positive experiences	0.006	(−0.013, 0.024)
Warm parenting	−0.005	(−0.013, 0.003)
Parental IPV	0.042	(−0.031, 0.110)
IPV*warm parenting	0.000	(−0.005, 0.006)
Co‐parenting alliance	−0.011	(−0.025, 0.004)
Parental IPV	0.037	(0.005, 0.069)
IPV*co‐parenting alliance	0.003	(−0.011, 0.017)
Relationship between parents	−0.002	(−0.005, 0.001)
Parental IPV	0.009	(−0.045, 0.062)
IPV*relationship between parents	0.002	(−0.000, 0.005)
Relationship with parents	−0.011	(−0.018, −0.005)
Parental IPV	0.017	(−0.110, 0.140)
IPV*relationship with parents	0.001	(−0.003, 0.004)
Relationship with teachers	−0.021	(−0.029, −0.013)
Parental IPV	0.016	(−0.066, 0.099)
IPV*relationship with teachers	0.002	(−0.004, 0.009)
Relationship with peers	−0.026	(−0.046, −0.007)
Parental IPV	0.020	(−0.074, 0.110)
IPV*relationship with peers	0.003	(−0.009, 0.015)
Positive home education environment	−0.003	(−0.008, 0.001)
Parental IPV	0.048	(0.001, 0.098)
IPV*positive home education environment	0.000	(−0.004, 0.004)
Out‐of‐home activities with parents	−0.009	(−0.017, −0.000)
Parental IPV	0.053	(0.001, 0.110)
IPV*out‐of‐home activities with parents	−0.001	(−0.008, 0.006)
Out‐of‐home activities without parents	−0.001	(−0.011, 0.010)
Parental IPV	0.057	(0.026, 0.087)
IPV*out‐of‐home activities without parents	−0.003	(−0.011, 0.005)
School enjoyment	−0.017	(−0.022, −0.012)
Parental IPV	0.011	(−0.078, 0.100)
IPV*school enjoyment	0.002	(−0.002, 0.006)
Neighbourhood safety and cohesion	−0.022	(−0.030, −0.015)
Parental IPV	0.029	(−0.083, 0.140)
IPV*neighbourhood safety and cohesion	0.001	(−0.005, 0.006)

*Note*: All models were adjusted for confounding (all measured at birth): child's sex, family's ethnicity, maternal partnership status, maternal and paternal education, maternal and paternal social class, financial difficulties, housing tenure, crowding index, mother smoking during pregnancy, mother drinking during pregnancy, mother's age at birth, parental mental health problems.

Abbreviations: b, Poisson beta coefficient; IPV, intimate partner violence; 95% CI, 95% confidence interval.

## DISCUSSION

There was strong evidence for the association between parental IPV as well as total positive experiences and depressive symptoms at age 18. Most positive experiences were linked with lower levels of depressive symptoms in the entire sample. Among those who were exposed to parental IPV, there was evidence for the association between total positive experiences, relationship with peers, school enjoyment, neighbourhood safety and cohesion with depressive symptoms. Relationships with parents, teachers and between parents were additionally associated with depressive symptoms among those without IPV. Despite some of the point estimates of the association between positive experiences and depressive symptoms appearing to differ between those with and without IPV, we did not find group differences based on the interaction test. This may be due to relatively wide confidence intervals around point estimates in the IPV exposed group. Moreover, as the strength of the associations between positive experiences and depressive symptoms was relatively small, detecting differences in these associations would require large statistical power.

Consistent with our findings, previous cross‐sectional research indicated that factors such as peer relationships, neighbourhood satisfaction, positive school experiences, were associated with better mental health both in those with and without childhood adversities such as maltreatment or abuse (Afifi et al., 2016; Afifi & MacMillan, 2011; Bellis et al., 2017; Cheung et al., 2017; Eisenberg et al., 2007; Hu et al., 2015; Hughes et al., 2018; Latham et al., 2021). Hence, these factors can serve as potential candidates for policy or practice interventions not only targeting those with IPV, but that could also be implemented in a more universal manner across general population of adolescents.

On the other hand, these studies also emphasised the importance of relationships with and between parent's mental health, which were not found to be linked with depressive symptoms among those with experiences of IPV. Likewise, other parental experiences, such as warm parenting, co‐parenting alliance and out‐of‐activities with parents appear to be linked with depressive symptoms among those without but not with IPV. This discrepancy in the associations may be due to IPV and parenting‐related factors being part of the same phenomenon, where positive experiences, as reflected by high scores, are cancelled out by exposure to IPV. Hence, effective strategies mitigating the harmful effects of IPV on mental health could focus on providing the adolescents with positive experiences outside their home environment, for instance, at school, community or through extracurricular activities. Including positive experiences as part of an intervention may also help to shift the narrative from being adversity‐centered to building resources, such as supportive relationships. Capacity‐building initiatives aimed at promoting resilience already exist, showing promising results, for instance, *Within My Reach* in the USA (Sterrett‐Hong et al., 2018) and *Be You* in Australia (Hoare et al., 2020).

### Strengths and limitations

The strengths of our study address key weaknesses of previous relevant research. Namely, we relied on a longitudinal design with prospective measures of all variables, with chronological order of IPV, positive experiences and depressive symptoms. Moreover, the availability of rich information on early life allowed us to account for a wide range of potentially confounding factors.

Despite the major strengths of our study, its findings are based on observational design and should not be treated as causal. The associations examined in the study rely on the assumption that differences between groups, other than due to exposure to IPV and/or positive experiences, are accounted for by adjusting for confounding. However, it is unlikely that all such confounding factors were included in the analyses. For instance, there may be genetic influences, such as Monoamine oxidase A, increasing the propensity of family members to engage in violence and suffer from mental health problems (Godar et al., 2016; Huang et al., 2004; Kim‐Cohen et al., 2006). However, adversities such as IPV are typically considered to have social roots, for example, due to poverty (Lacey et al., 2022), which is also linked with mental health, and we controlled for a range of socioeconomic factors. As IPV cannot be randomised due to ethical reasons, our study still presents strong candidates for positive experiences that can be targeted by public mental health interventions aiming to improve mental health of individuals with the experience of IPV.

The second important limitation of our study is the generalisability of its findings. The most socioeconomically disadvantaged families, as well as ethnic minorities, are somewhat underrepresented in ALSPAC compared to general population. This bias is exacerbated by attrition and non‐response, both of which were found to be greater among non‐white and socioeconomically disadvantaged population. This is likely to result in underestimation of IPV and depressive symptoms and to overestimate positive experiences. We would also expect the association between IPV and depressive symptoms to be weaker in the study sample, compared with the general population, and stronger for positive experiences. This was found in our comparison between unadjusted estimates in the complete cases and the imputed sample. Finally, our findings, as is typically the case with observational studies, relied on self‐reports of IPV and most of the positive experiences by parents. The reports of IPV can be influenced by guilt or shame, whereas accounts of positive experiences might be affected by the desire to favourably portray oneself (Loxton et al., 2017).

## CONCLUSION

Most positive experiences were linked with lower levels of depressive symptoms in the entire study sample. However, among those with IPV, this association was found only for relationship with peers, school enjoyment, neighbourhood safety and cohesion on depressive symptoms. The key implication of our study is that strategies providing adolescents with positive experiences outside their home environment, for instance, at school, community or through extracurricular activities may help to improve mental health of children with IPV, hence potentially mitigating its consequences.

## AUTHOR CONTRIBUTIONS

**Dawid Gondek**: Conceptualization; Formal analysis; Investigation; Methodology; Writing – original draft; Writing – review & editing. **Gene Feder**: Conceptualization; Funding acquisition; Supervision; Writing – review & editing. **Laura D. Howe**: Methodology; Supervision; Writing – review & editing. **Ruth Gilbert**: Conceptualization; Funding acquisition; Supervision; Writing – review & editing. **Emma Howarth**: Conceptualization; Funding acquisition. **Jessica Deighton**: Conceptualization; Writing – review & editing. **Rebecca E. Lacey**: Conceptualization; Supervision; Writing – review & editing.

## CONFLICTS OF INTEREST

The authors have declared that they have no competing or potential conflicts of interest.

## ETHICAL CONSIDERATIONS

Ethical approval was obtained from the ALSPAC Ethics and Law Committee and the Local Research Ethics Committee. Informed consent was provided from all participants or their carers for each data collection.

## Supporting information

Supporting Information S1

## Data Availability

ALSPAC data access is through a system of managed open access. The procedure for accessing data can be found on the ALSPAC website: http://www.bristol.ac.uk/alspac/. If you have any questions about accessing data, please email alspac‐data@bristol.ac.uk.
