# Supplementary material for: Factors mitigating the harmful effects of intimate partner violence on adolescents' depressive symptoms—A longitudinal birth cohort study
Source: JCPP Adv. 2023 Jan 25;3(1):e12134. doi: 10.1002/jcv2.12134 (PMC10241470; doi:10.1002/jcv2.12134)
Supplement: Supplementary file 1 — Supporting Information S1 [file JCV2-3-e12134-s001.docx]

# Supporting Information


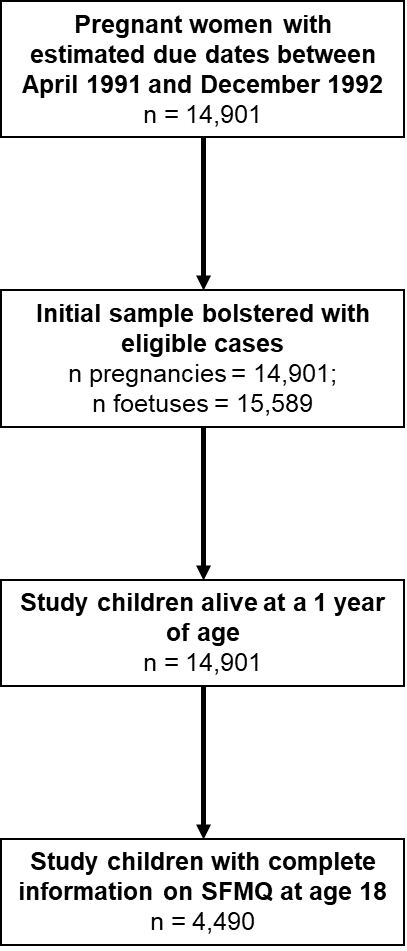


**Figure S1. Sample flow diagram.**

| **Table S1. Details about measurement of positive experiences.** | | | | |
| --- | --- | --- | --- | --- |
| **Exposures** | **Measurement items** | **Response option** | **Respondent** | **Age assessed** |
| Parental intimate partner violence (IPV) | Partner's partner was physically cruel to them since study child was XX old | Yes & affected me a lot/Yes, moderately affected/Yes, mildly affected/Yes, but did not affect me at all (1), No did not happen (0) | Mother, partner | 2yrs9m, 3yrs11m, 5yrs1m, 6yrs1m, 9yrs2m |
|  | Whether parter was emotionally cruel to mum since study child was XX old and effect this had |  |  |  |
| Warm parenting | Frequency that mother or other adult female/partner cuddles child | Nearly everyday (4), 2-5 times per week (3), Once per week (2), <once a week (1), Never (0) | Mother, partner | 11yrs8m |
|  | Frequency that mother or other adult female/partner kisses child goodnight |  |  |  |
| Co-parenting alliance | Mother's partner/mother provides the emotional support mother needs | Exactly feel (3), Often feel (2), Sometimes feel (1), Never feel (0) | Mother, partner | 12yrs1m |
|  | Mother/partner can rely on partner/mother to take over when mother is tired |  |  |  |
| Relationships between parents | Partner/mother is very considerate of mother/partner | Very true (3), Moderately true (2), Somewhat true (1), Not at all true (0) | Mother, partner | 12yrs1m |
|  | Partner/mother is a good companion to mother/partner |  |  |  |
|  | Partner/mother is affectionate to mother/partner |  |  |  |
|  | Partner/mother confides closely in mother/partner |  |  |  |
|  | Partner/mother understands mother's/partner's problems and worries |  |  |  |
|  | Partner/mother is physically gentle and considerate to mother/partner |  |  |  |
|  | Partner/mother makes mother/partner feel needed |  |  |  |
|  | Partner/mother is very loving to mother/partner |  |  |  |
|  | Partner/mother is fun to be with |  |  |  |
|  | Partner/mother shows appreciation of mother/partner |  |  |  |
|  | Partner/mother is gentle and kind to mother/partner |  |  |  |
|  | Partner/mother speaks to mother in a warm and friendly voice |  |  |  |
| Relationship with parents | Study child gets along well with parent(s) | Not true (1), Mostly untrue (2), Partly true (3), Mostly true (4), True (5) | Child | 9yrs7m |
|  | Study child is understood by parent(s) |  |  |  |
|  | Study child likes their parent(s) |  |  |  |
|  | Study child is liked by parent(s) |  |  |  |
|  | Parent(s) and study child spend a lot of time together |  |  |  |
|  | Study child's parent(s) is easy to talk to |  |  |  |
|  | Study child gets along well with parent(s) |  |  |  |
|  | Study child and parent(s) have a lot of fun together |  |  |  |
| Relationship with teachers | Child's school is a place where their teacher is fair to them | Agree (3), Mostly agree (2), Mostly disagree (1), Disagree (0) | Child | 11yrs2m |
|  | Child's school is a place where their teacher takes an interest in helping them with their work |  |  |  |
|  | Child's school is a place where their teacher listens to what they say |  |  |  |
|  | Child's school is a place where their teacher helps them to do their best |  |  |  |
|  | Child's school is a place where their teacher treats them fairly in class |  |  |  |
| Relationship with peers | Happy with no. of friends | Very happy (3), Quite happy (2), Quite unhappy (1), Unhappy (0) | Child | 10yrs6m |
|  | Child can talk to friends about problems | Most of time (3), Sometimes (2), Not often (1), Not at all (0) |  |  |
|  | Friends understand child | Most of time (3), Sometimes (2), Not often (1), Not at all (0) |  |  |
| Positive home education environment | Frequency that mother or other adult female/partner makes things with child | Agree (3), Mostly agree (2), Mostly disagree (1), Disagree (0) | Mother | 11yrs8m |
|  | Frequency that mother or other adult female/partner sings with child |  |  |  |
|  | Frequency that mother or other adult female/partner reads to/with child |  |  |  |
|  | Frequency that mother or other adult female/partner play with toys with child |  |  |  |
|  | Frequency that mother or other adult female/partner does active play with child |  |  |  |
|  | Frequency that mother or other adult female/partner draws or paints with child |  |  |  |
| Out-of-home activities with parents | Frequency that mother or other adult female/partner goes with child to park or play | Nearly everyday (4), 2-5 times per week (3), Once per week (2), <once a week, Never (0) | Mother | 11yrs8m |
|  | Frequency that mother or other adult female/partner takes child swimming |  |  |  |
|  | Frequency that mother or other adult female/partner takes child shopping |  |  |  |
|  | Frequency that mother or other adult female/partner takes child to watch sport |  |  |  |
| Out-of-home activities without parents | Child went to a playground without an adult last weekend | No (0), Yes (1) | Child | 13yrs |
|  | Child went to a park/playing field without an adult last weekend |  |  |  |
|  | Child went swimming without an adult last weekend |  |  |  |
|  | Child played outside their home without an adult last weekend |  |  |  |
|  | Child hung around in the street without an adult last weekend |  |  |  |
|  | Child went for a walk without an adult last weekend |  |  |  |
|  | Child cycled around without an adult last weekend |  |  |  |
|  | Child went to the shops without an adult last weekend |  |  |  |
|  | Child went to the library without an adult last weekend |  |  |  |
|  | Child went to a club/class without an adult last weekend |  |  |  |
|  | Child went to a leisure centre without an adult last weekend |  |  |  |
|  | Child went to the cinema without an adult last weekend |  |  |  |
|  | Child went to a football/sports match without an adult last weekend |  |  |  |
|  | Child played in a football/sports match without an adult last weekend |  |  |  |
| School enjoyment | Child's school is a place where they really like to go each day | Agree (3), Mostly agree (2), Mostly disagree (1), Disagree (0) | Child | 11yrs2m |
|  | Child's school is a place where they like to be |  |  |  |
|  | Child's school is a place where they feel happy |  |  |  |
|  | Child's school is a place where learning is fun |  |  |  |
|  | Child's school is a place where they have a lot of fun |  |  |  |
|  | Child's school is a place where they enjoy what they do in class |  |  |  |
|  | Child's school is a place where they get excited about the work they do |  |  |  |
|  | Child's school is a place where the work they do is interesting |  |  |  |
|  | Child's school is a place where they get enjoyment |  |  |  |
|  | Child's school is a place where they feel happy with the standard of their work |  |  |  |
| Neighbourhood safety and cohesion | Respondent usually feels safe in their neighbourhood | Strongly agree (3), Agree (2), Disagree (1), Strongly disagree (0) | Child | 14yrs1m |
|  | Respondent would feel safe if at home alone |  |  |  |
|  | Respondent has stopped to talk to someone on the street in their neighbourhood in the past month |  |  |  |
|  | Respondent is scared of some of the people in their neighbourhood |  |  |  |
|  | Respondent is scared of being home when no adults are there |  |  |  |
|  | Respondent wouldn't mind if they had to move from here to some other neighbourhood |  |  |  |
|  | Respondent on the whole is happy living in their neighbourhood |  |  |  |
|  | People in respondent's neighbourhood look out for each other |  |  |  |
|  | Respondent knows most of the people in their neighbourhood |  |  |  |

## **Appendix S1. Analytical approach to missing data.**

The missing values were replaced with multiple imputation. In line with the recommendations, we included all the variables from the analysis in the imputation model to preserve the relationship between the variables (Moons et al., 2006; White et al., 2011). To ensure that the model is compatible with the analysis, the interaction terms were included (Tilling et al., 2016), treating them as “just another variable” (the JAV approach) (Seaman et al., 2012).

The multiple imputation works under the missing at random (MAR) assumption (Collins et al., 2001; Richards et al., 1989). The MAR mechanism, which is largely untestable, implies that systematic differences between the missing and the observed values can be explained by observed data (Collins et al., 2001). The missing-at-random assumption is largely untestable (Molenberghs et al., 2008). Hence, we also enriched the imputation model and further maximised the plausibility of the MAR assumption with birthweight as an auxiliary variable. This variable was not part of the substantive model of interest, but it was associated with missingness and with depressive symptoms. Including auxiliary variables can improve the accuracy of the MI and minimise non-random variation in the imputed values (Sterne et al., 2009). In addition, the imputation model was rich due to including covariates used in the analyses, such as socioeconomic indicators or mental health in childhood, which were predictive of both missingness (see eTable 3) and depressive symptoms.

The missing data were imputed using multiple imputation by chained equations (MICE), due to the non-monotone pattern of missing values, and due to its ability to accommodate various types of variables in the imputation model, including continuous and categorical ones. This approach uses a series of univariate conditional imputation models to impute missing data (van Buuren, 2007). Continuous variables were imputed using predictive mean matching and categorical ones using logistic regressions. The predictive mean matching approach provides robust estimates if the normality assumption is in question (Morris et al., 2014), this is particularly relevant to mental health outcomes as they tend to be skewed (Counsell et al., 2011), or when associations are non-linear (Morris et al., 2014).

**References**

Collins, L. M., Schafer, J. L., & Kam, C. M. (2001). A comparison of inclusive and restrictive strategies in modern missing data procedures. In *Psychological Methods*. https://doi.org/10.1037/1082-989x.6.4.330

Counsell, N., Cortina-Borja, M., Lehtonen, A., & Stein, A. (2011). Modelling psychiatric measures using Skew-Normal distributions. *European Psychiatry*. https://doi.org/10.1016/j.eurpsy.2010.08.006

Molenberghs, G., Beunckens, C., Sotto, C., & Kenward, M. G. (2008). Every missingness not at random model has a missingness at random counterpart with equal fit. *Journal of the Royal Statistical Society. Series B: Statistical Methodology*. https://doi.org/10.1111/j.1467-9868.2007.00640.x

Moons, K. G. M., Donders, R. A. R. T., Stijnen, T., & Harrell, F. E. (2006). Using the outcome for imputation of missing predictor values was preferred. *Journal of Clinical Epidemiology*. https://doi.org/10.1016/j.jclinepi.2006.01.009

Morris, T. P., White, I. R., & Royston, P. (2014). Tuning multiple imputation by predictive mean matching and local residual draws. *BMC Medical Research Methodology*. https://doi.org/10.1186/1471-2288-14-75

Richards, L. E., Little, R. J. A., & Rubin, D. B. (1989). Statistical Analysis with Missing Data. *Journal of Marketing Research*. https://doi.org/10.2307/3172915

Seaman, S. R., Bartlett, J. W., & White, I. R. (2012). Multiple imputation of missing covariates with non-linear effects and interactions: An evaluation of statistical methods. *BMC Medical Research Methodology*. https://doi.org/10.1186/1471-2288-12-46

Sterne, J. A. C., White, I. R., Carlin, J. B., Spratt, M., Royston, P., Kenward, M. G., Wood, A. M., & Carpenter, J. R. (2009). Multiple imputation for missing data in epidemiological and clinical research: potential and pitfalls. *BMJ*, *338*.

Tilling, K., Williamson, E. J., Spratt, M., Sterne, J. A. C., & Carpenter, J. R. (2016). Appropriate inclusion of interactions was needed to avoid bias in multiple imputation. *Journal of Clinical Epidemiology*. https://doi.org/10.1016/j.jclinepi.2016.07.004

van Buuren, S. (2007). Multiple imputation of discrete and continuous data by fully conditional specification. *Statistical Methods in Medical Research*. https://doi.org/10.1177/0962280206074463

White, I. R., Royston, P., & Wood, A. M. (2011). Multiple imputation using chained equations: Issues and guidance for practice. *Statistics in Medicine*, *30*(4), 377–399.

| **Table S2. The proportion of missing information in each variable (n=4,490).** | | |
| --- | --- | --- |
| Variable (age in years when measured) | N missing | % missing |
| ***Outcome*** |  |  |
| Depressive symptoms (18) | 0 | 0.0 |
|  |  |  |
| ***Exposures*** |  |  |
| Parental intimate partner violence (3) | 668 | 14.9 |
| Parental intimate partner violence (4) | 699 | 15.6 |
| Parental intimate partner violence (5) | 797 | 17.8 |
| Parental intimate partner violence (6) | 867 | 19.3 |
| Parental intimate partner violence (7) | 867 | 19.3 |
| Parental intimate partner violence (8) | 821 | 18.3 |
| Warm parenting (12) | 934 | 20.8 |
| Co-parenting alliance (12) | 902 | 20.1 |
| Relationships between parents (12) | 1,235 | 27.5 |
| Relationship with parents (9.5) | 977 | 21.8 |
| Relationship with teachers (11) | 729 | 16.2 |
| Relationship with peers (10.5) | 716 | 16.0 |
| Positive home education environment (11.5) | 1,087 | 24.2 |
| Out-of-home activities with parents (11.5) | 1,064 | 23.7 |
| Out-of-home activities without parents (13) | 798 | 17.8 |
| School enjoyment (11) | 1,062 | 23.7 |
| Neighbourhood safety and cohesion (14) | 2,208 | 49.2 |
|  |  |  |
| ***Confounding factors*** |  |  |
| Child’s sex | 0 | 0.0 |
| Maternal social class (0) | 807 | 18.0 |
| Paternal social class (0) | 1,155 | 25.7 |
| Maternal education (0) | 378 | 8.4 |
| Paternal education (0) | 475 | 10.6 |
| Family’s ethnicity | 433 | 9.6 |
| Financial difficulties (0) | 492 | 11.0 |
| Mother smoking during pregnancy (0) | 327 | 7.3 |
| Mother drinking during pregnancy (0) | 408 | 9.1 |
| Mother’s age at birth (0) | 517 | 11.5 |
| Housing tenure (0) | 363 | 8.1 |
| Crowding index (0) | 402 | 9.0 |
| Maternal partnership status (0) | 327 | 7.3 |
| Parental mental health problems (0) | 376 | 8.4 |
|  |  |  |
| ***Auxiliary variables*** |  |  |
| Birthweight | 296 | 6.6 |

| **Table S3. Predictors of having missing information on any exposure in the study sample (n=4,490).** | | | | |
| --- | --- | --- | --- | --- |
| Exposures (range; age in years when measured) | Risk ratio | | 95% CI | |
| Depressive symptoms (0-26; 18) | 1.01 | 1.00 | | 1.01 |
| Parental intimate partner violence (0-6; 1.5-8) | 1.05 | 1.02 | | 1.07 |
| Total positive experiences (0-11; 9.5-14) | 1.02 | 0.95 | | 1.10 |
| Maternal social class (0) |  |  | |  |
| I/II (reference) |  |  | |  |
| III non-manual/III manual | 1.02 | 0.98 | | 1.07 |
| IV/V | 1.13 | 1.08 | | 1.19 |
| Maternal education (0) |  |  | |  |
| Degree (reference) |  |  | |  |
| A level | 1.11 | 1.05 | | 1.18 |
| CSE/Vocational/O level | 1.15 | 1.09 | | 1.21 |
| Family's ethnicity |  |  | |  |
| White (reference) |  |  | |  |
| Non-white | 1.15 | 1.08 | | 1.23 |
| Financial difficulties (0-15; 0) | 1.01 | 1.01 | | 1.02 |
| Mother smoking during pregnancy (0) |  |  | |  |
| No (reference) |  |  | |  |
| Yes | 1.13 | 1.09 | | 1.18 |
| Parental mental health problems (0) |  |  | |  |
| No (reference) |  |  | |  |
| Yes | 1.12 | 1.07 | | 1.18 |
| *Note.* CSE = Certificate of Secondary Education; O level = Ordinary level; A level = Advanced level; 95% CI = 95% confidence interval. | | | | |

| **Table S4. Descriptive information about the studied variables – full available sample for each variable.** | | | | | |  |
| --- | --- | --- | --- | --- | --- | --- |
| Variable (age in years when measured) | N total | Mean | SD | Min | Max |  |
| ***Outcome*** |  |  |  |  |  |  |
| Depressive symptoms (18) | 4,490 | 6.59 | 5.25 | 0 | 26 |  |
|  |  |  |  |  |  |  |
| ***Exposures*** |  |  |  |  |  |  |
| Parental intimate partner violence (1.5 – 11) | 5,757 | 0.39 | 0.93 | 0 | 6 |  |
| Total positive experiences (9-5 – 14) | 1,719 | 3.27 | 1.96 | 0 | 10 |  |
| Warm parenting (12) | 7,105 | 13.35 | 3.43 | 0 | 16 |  |
| Co-parenting alliance (12) | 7,132 | 3.19 | 1.93 | 0 | 6 |  |
| Relationships between parents (12) | 6,375 | 26.11 | 8.58 | 0 | 36 |  |
| Relationship with parents (9.5) | 7,512 | 37.51 | 3.85 | 8 | 40 |  |
| Relationship with teachers (11) | 7,897 | 12.54 | 3.16 | 0 | 25 |  |
| Relationship with peers (10.5) | 7,296 | 7.86 | 1.36 | 0 | 9 |  |
| Positive home education environment (11.5) | 6,772 | 13.29 | 6.72 | 0 | 48 |  |
| Out-of-home activities with parents (11.5) | 6,845 | 8.48 | 3.67 | 0 | 32 |  |
| Out-of-home activities without parents (13) | 6,898 | 3.08 | 2.81 | 0 | 14 |  |
| School enjoyment (11) | 7,108 | 21.89 | 5.46 | 0 | 30 |  |
| Neighbourhood safety and cohesion (14) | 3,992 | 22.26 | 3.92 | 3 | 30 |  |
|  |  |  |  |  |  |  |
| ***Confounding factors*** |  |  |  |  |  |  |
| Financial difficulties (0) | 12,148 | 2.91 | 3.54 | 0 | 15 |  |
| Mother's age during birth (0) | 12,060 | 28.64 | 4.86 | 15 | 46 |  |
|  | N total | N | % |  |  |  |
| Child’s sex | 15,038 |  |  |  |  |  |
| Female |  | 7,348 | 48.9 |  |  |  |
| Male |  | 7,690 | 51.1 |  |  |  |
| Family’s ethnicity | 12,136 |  |  |  |  |  |
| White |  | 11,523 | 94.9 |  |  |  |
| Non-white |  | 613 | 5.1 |  |  |  |
| Maternal marital status (0) | 13,544 |  |  |  |  |  |
| Never married |  | 2,595 | 19.2 |  |  |  |
| Widowed/divorced/separated |  | 816 | 6.0 |  |  |  |
| 1st marriage |  | 9,252 | 68.3 |  |  |  |
| 2nd/3rd marriage |  | 881 | 6.5 |  |  |  |
| Maternal education (0) | 12,478 |  |  |  |  |  |
| Degree |  | 1,608 | 12.9 |  |  |  |
| A level |  | 2,793 | 22.4 |  |  |  |
| CSE/Vocational/O level |  | 8,077 | 64.7 |  |  |  |
| Paternal education (0) | 11,996 |  |  |  |  |  |
| Degree |  | 2,178 | 18.2 |  |  |  |
| A level |  | 3,115 | 26.0 |  |  |  |
| CSE/Vocational/O level |  | 6,703 | 55.9 |  |  |  |
| Maternal social class (0) | 11,112 |  |  |  |  |  |
| I/II |  | 3,519 | 31.7 |  |  |  |
| III non-manual/III manual |  | 5,177 | 46.6 |  |  |  |
| IV/V |  | 2,416 | 21.7 |  |  |  |
| Paternal social class (0) | 9,517 |  |  |  |  |  |
| I/II |  | 3,918 | 41.2 |  |  |  |
| III non-manual/III manual |  | 4,760 | 50.0 |  |  |  |
| IV/V |  | 839 | 8.8 |  |  |  |
| Housing tenure (0) | 13,486 |  |  |  |  |  |
| Mortgaged/owned |  | 9,871 | 73.2 |  |  |  |
| Not owned |  | 3,615 | 26.8 |  |  |  |
| Crowding index (0) | 13,246 |  |  |  |  |  |
| <= 0.5 |  | 5,484 | 41.4 |  |  |  |
| >0.5 - 0.75 |  | 4,161 | 31.4 |  |  |  |
| >0.75 – 1 |  | 2,679 | 20.2 |  |  |  |
| > 1 |  | 922 | 7.0 |  |  |  |
| Mother smoking during pregnancy (0) | 13,344 |  |  |  |  |  |
| No |  | 9,981 | 74.8 |  |  |  |
| Yes |  | 3,363 | 25.2 |  |  |  |
| Mother drinking during pregnancy (0) | 12,429 |  |  |  |  |  |
| No |  | 3,732 | 30.0 |  |  |  |
| Yes |  | 8,697 | 70.0 |  |  |  |
| Parental mental health problems (0) | 12,400 |  |  |  |  |  |
| No |  | 10,840 | 87.4 |  |  |  |
| Yes |  | 1,560 | 12.6 |  |  |  |
| *Note.* CSE = Certificate of Secondary Education; O level = Ordinary level; A level = Advanced level; 95% CI = 95% confidence interval; SD = standard deviation. | | | | | | |

| **Table S5. Estimates of the association between each exposure and depressive symptoms at age 18 – using a sample with complete information on parental intimate parental violence, all positive experiences, and the measure of psychopathology.** | | |
| --- | --- | --- |
| N = 1,015 | Unadjusted estimates  (Poisson coefficient) | |
| Exposures (range; age in years when measured) | b | (95% CI) |
| Parental intimate partner violence (0-6; 1.5-11) | 0.110 | (0.051, 0.170) |
| Total positive experiences (0-11; 9.5-14) | -0.046 | (-0.074, -0.018) |
| Warm parenting (0-16; 12) | -0.002 | (-0.018, 0.014) |
| Co-parenting alliance (0-6; 12) | -0.021 | (-0.054, 0.011) |
| Relationships between parents (0-36; 12) | -0.002 | (-0.009, 0.004) |
| Relationship with parents (8-40; 9.5) | -0.012 | (-0.024, -0.001) |
| Relationship with teachers (0-25; 11) | -0.033 | (-0.049, -0.017) |
| Relationship with peers (0-9; 10.5) | -0.020 | (-0.066, 0.026) |
| Positive home education environment (0-48; 11.5) | -0.007 | (-0.017, 0.003) |
| Out-of-home activities with parents (0-32; 11.5) | -0.018 | (-0.035, -0.001) |
| Out-of-home activities without parents (0-14; 13) | -0.007 | (-0.028, 0.015) |
| School enjoyment (0-30; 11) | -0.024 | (-0.034, -0.014) |
| Neighbourhood safety and cohesion (3-30; 14) | -0.034 | (-0.049, -0.020) |
| *Note.* IPV = intimate partner violence; b = Poisson beta coeffiecient; 95% CI = 95% confidence interval. | | |
